# Indoor levels of volatile organic compounds and formaldehyde from emission sources at elderly care centers in Korea

**DOI:** 10.1371/journal.pone.0197495

**Published:** 2018-06-07

**Authors:** Kyoungho Lee, Jae-Hyun Choi, Seokwon Lee, Hee-Jin Park, Yu-Jin Oh, Geun-Bae Kim, Woo-Seok Lee, Bu-Soon Son

**Affiliations:** 1 Samsung Health Research Institute, Samsung Electronics Co., Ltd., Hwaseong, Republic of Korea; 2 Department of Environmental Health Science, Soonchunhyang University, Asan, Republic of Korea; 3 Environmental Health Research Division, National Institute of Environment Research, Incheon, Republic of Korea; Telethon Institute for Child Health Research, AUSTRALIA

## Abstract

The objective of this study is to characterize indoor and outdoor levels of volatile organic compounds (VOCs) and formaldehyde (HCHO) and identify indoor emission sources in thirty elderly care centers (ECCs) located in the Seoul metropolitan city and Gyeonggi province in Korea. Air monitoring samples from indoor and outdoor environments were collected from January to December in 2007. Statistical analyses of indoor and outdoor VOCs and HCHO levels in three rooms (a bedroom, living, and dining rooms) of each ECC were performed, and these were compared to identify environmental factors associated with an increase of indoor pollution levels. Total volatile organic compounds (TVOC) levels were significantly (*p*<0.05) different between indoor (230.7±1.7 μg/m^3^) and outdoor (137.8±1.9 μg/m^3^) environments, with an I/O ratio of 1.67. The indoor HCHO level (20.1±1.6 μg/m^3^) was significantly (*p*<0.05) higher than the outdoor level (8.1±1.9 μg/m^3^), with an I/O ratio of 2.48. Indoor VOCs and HCHO levels in the bedrooms were significantly (*p*<0.05) higher than those in the living and dining rooms. Furthermore, indoor levels of VOCs and HCHO at ECCs were significantly (*p*<0.05) different depending on environmental factors such as the use of carpet, paint, and wooden furniture. In multiple regression analysis, indoor VOCs and HCHO levels at ECCs were significantly (*p*<0.05) correlated with two micro-environmental factors: the use of carpet and paint. This study confirmed that indoor VOCs and HCHO levels were significantly higher than those in outdoor environments. These air pollutants were mainly emitted from indoor sources, such as carpet, paint, and construction materials at the ECCs in Korea.

## Introduction

Levels of indoor air pollutants are increasing; therefore, concerns over indoor air quality (IAQ) and its potential health effects have increased within the scientific community in the field of environmental health sciences. The pollutants implicated include volatile organic compounds (VOCs), environmental tobacco smoke (ETS), particulate matter (PM), nitrogen oxides (NOx), carbon monoxide (CO), and ozone (O_3_) emitted mostly from building materials, new furniture, fresh paint, combustion processes, and consumer products (cleaners, solvents, mothballs, etc.). Their levels are significantly higher in some indoor environments than outdoor environments. It has been also shown that long-term exposure to low-level indoor air pollutants could produce adverse health effects including reduced lung function, oxidative stress, acute pulmonary symptom, and incidence of asthma, allergic rhinitis, and other respiratory diseases among susceptible populations, including children, pregnant women, and the elderly [1–5].

A number of studies have shown that indoor levels of various types of air pollutants, such as volatile organic compounds (VOCs), formaldehyde (HCHO), carbon monoxide (CO), carbon dioxide (CO_2_), ozone (O_3_), aldehydes, and fine particulate matter (PM) in modern residential buildings are significantly higher than their outdoor levels for several reasons. These reasons include the existence of indoor emission sources (e.g., paint and associated supplies, household furnishings, consumer products, combustion appliances, etc.), low ventilation rates, environmental tobacco smoking (ETS), internal fuel combustion for cooking, seasonal variations in temperature and relative humidity, and other environmental conditions [6–8].

Park et al. found that indoor levels of TVOC (from 120 to 328 μg/m^3^) and HCHO (from 88 to 134 μg/m^3^), mainly emitted from wooden materials, were significantly higher in new residential buildings than in older ones. Therefore, they concluded that the indoor levels of VOCs and HCHO in newer residences were significantly higher than ones at older residences, and a decreasing tendency of indoor air pollution levels in newer residences was more influenced by the aging decreases of emission sources rather than ventilation systems or its efficiency over three years [9]. Kim et al. also found a significant increase in the levels of TVOC (from 517 to 1,920 μg/m^3^) and HCHO (from 209 to 457 μg/m^3^) in new buildings, and these increases were associated with the use of building materials (adhesives, coatings, or paint) and the purchase of new furniture. They concluded that the levels of TVOC and HCHO exceeded all existing recommended levels found in domestic and international guidelines, which indicates that these air pollutants were primarily emitted from indoor environments, not outdoor environments [10]. Kabir et al. observed that no level of indoor air pollutants exceeded a Korean guideline in child care buildings, medical buildings, or elementary schools, but some of pollutants (HCHO, PM_10_ and bioaerosol) were relatively higher in the summer season than in other seasons [11]. Previous studies have found that the levels of air pollutants measured in various micro-environments, such as living rooms, dining rooms, and bedrooms in Korean elderly care centers (ECCs) are higher than the reference limit values for several chemical and biological pollutants, such as VOCs, HCHO, CO, CO_2_, O_3_, bacteria, and fungi established by the Ministry of Environment (MoE)[12, 13]. However, the authors found that the general population and elderly people spend approximately 80~90% of their daily time indoors (in homes, offices, shops, public facilities, etc.), with poor ventilation rates and irregular seasonal factors (i.e., higher temperature and relative humidity)[14–16]

In order to quantitatively characterize the levels of indoor and outdoor exposures in public facilities and evaluate their adverse health effects on susceptible populations including, children and the elderly, similar studies on indoor air quality (IAQ) in Korea have been conducted [17, 18]. The authors have found that indoor levels of air pollutants in ECCs are significantly increased because these air pollutants are mostly emitted from indoor sources in these facilities. Seo et al. have conducted a study of exposure to indoor air pollutants to identify such things as VOCs, HCHO, CO, CO_2_, PM, and total suspended bacteria (TSB) in 91 public facilities at Honam province in Korea from 2004 to 2005. According to their findings, all levels of air pollutants were less than reference limit values. However, a further study is needed to identify indoor emission sources and determine ways to improve IAQ at those public facilities [19]. Park et al. also found that at several child and elderly care centers measured in 2010, indoor levels of PM and CO_2_ were significantly higher than those in other residences or public facilities. They concluded that robust air cleaning and mechanical ventilation systems should be used to reduce indoor pollution levels because indoor levels of carbon dioxide at two ECCs in Incheon city of Korea exceeded the reference limit value of 1,000 ppm [20].

Son et al. conducted an exposure assessment study to collect indoor VOCs samples and evaluate personal VOCs exposure levels among sixty voluntary participants recruited from local communities in two metropolitan cities (i.e., Asan and Seoul) of Korea in 2001. Their study concluded that indoor levels of toluene in Seoul metropolitan city were approximately a hundred times higher than those in Asan city, while indoor VOCs pollution levels were also significantly higher than outdoor levels in both cities. Most importantly, their study provided evidence that personal VOCs exposures varied among different micro-environments of ECCs. Daily activity patterns of occupation, working condition, or time spent inside residences had a direct effect on them. Indoor VOC levels and personal exposures were closely associated with housing characteristics, such as the age of the building, indoor smoking, and types of residential buildings. However, previous studies failed to characterize VOCs or HCHO levels in indoor and outdoor environments. They did not statistically compare levels of air pollutants by each micro-environment, environmental factor, or construction year, or identify specific emission sources in the indoor environments of ECCs located in urban and suburban areas [21].

Because there was still a lack of evidence in the previous studies, a study was required to verify a hypothesis that indoor levels of air pollutants were not affected by outdoor air or environments and that these pollutants were primarily emitted from the indoor environments. This was done by calculating indoor-to-outdoor ratios (I/O ratios) of each pollutant collected inside and outside ECCs in Korea.

Therefore, the objective of the present study is to determine the levels of VOCs and HCHO inside and outside thirty ECCs located in Seoul metropolitan city and Gyeonggi province in Korea and compare indoor and outdoor pollution levels to identify indoor emission sources at the ECCs. A determination was also made of environmental factors that significantly increased indoor VOCs and HCHO levels in the target facilities.

## Materials and methods

### Study sites

**Fig 1:**
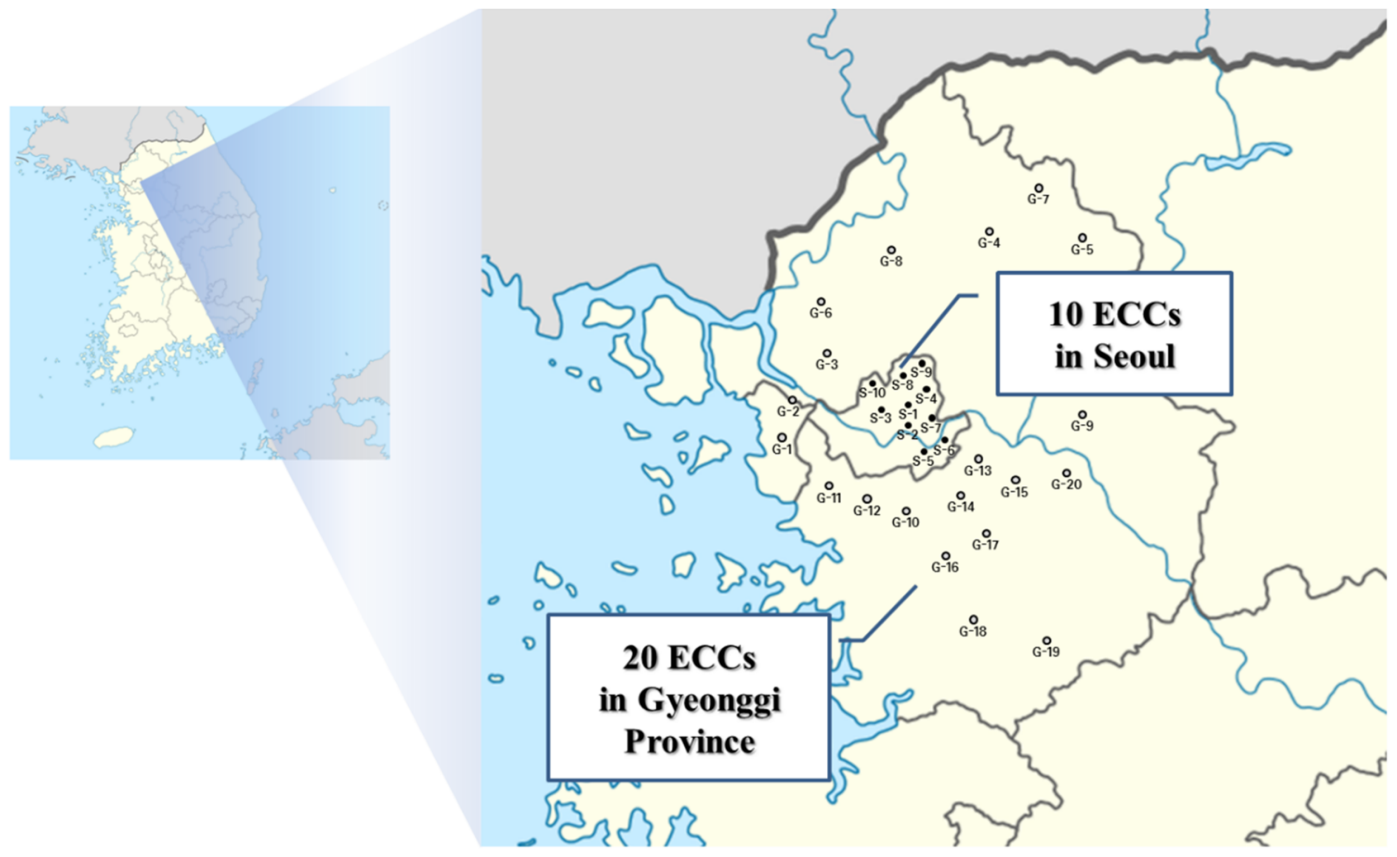
Geographical information on total 30 ECCs located in Seoul metropolitan city and Gyeonggi province in Korea.

A total of 30 ECCs located in Seoul metropolitan city (N = 10) and Gyeonggi province (N = 20) of Korea were selected to characterize levels of indoor air pollutants VOCs and HCHO in this study (Fig 1). We collected environmental samples in fixed locations of each indoor room (bedroom, living room, and dining room) at each ECC and outdoor environment on the first day of each quarter (1Q: January, 2Q: April, 3Q: July, 4Q: October) in 2007. Six teams began collecting indoor and outdoor samples of VOCs and HCHO at each ECC simultaneously at 7:00 a.m. and continued for one hour. Then they moved to different sites (i.e., ECCs) for the next sampling. At five different locations (i.e., ECCs), each team implemented air samplings until 6:00 p.m. of that day (i.e., one team collected indoor and outdoor monitoring samples at five ECCs for one day). Temperature (°C) and relative humidity (%) in both indoor and outdoor environments were measured on the same sampling days of each quarter. Average values of these environmental factors were calculated. In general, heating, ventilation, and air conditioning (HVAC) systems are installed in both the living rooms and the bedrooms of the most ECCs. General ventilation is implemented at all ECCs. However, we did not collect detailed information on the efficiency or specifications of HVAC systems installed. We did not measure exact air exchange rates at those ECCs because the purpose of this study was to characterize indoor inhalation exposure to both VOCs and HCHO, compare indoor and outdoor levels of VOCs and HCHO, and investigate the relationship between indoor levels of air pollutants and environmental factors at ECCs. All ECCs were located in urban and rural areas, the most of the elderly residents spent more than 90% of their daily activities in indoor environments. All of the study sites for measurement of air pollutants and sampling were authorized by the Korea Ministry of Environment (MoE).

### Sample collection

We collected air monitoring samples for VOCs on Tenax-TA sorbent tubes packed with 200 mg of adsorbent (60/80 mesh, Supelco, USA) for one hour using low-volume mini pumps (MP-Σ30H, Sibata, Japan) at a flow rate of 100ml/min. HCOH was measured using 2,4-dinitrophenylhydrazine(DNPH)-coated silica gel cartridges (Supelco, USA) with ozone scrubbers (Waters, USA) for one hour and low-volume pumps (MP- Σ100H, Sibata, Japan) at flow rate of 500ml/min. These pumps were calibrated to set the air flow rate. They were checked daily before and after each sampling. All collected samples were stored below 4°C in 50ml vials with inner coatings of aluminum. To determine if cross-contamination occurred between samples during transfer and storage, field and lab blank samples were also collected.

### Analytical methods

We used a standard solution which was the “Japanese indoor air standard mix” (Supelco, USA) including 52 chemicals. And 43 out of 52 chemicals were detected. We analyzed six substances (benzene, toluene, ethylbenzene, o,m,p-xylene, styrene and formaldehyde) among a total of 43 types of VOCs (hexane, chloroform, n-heptane, etc.) in the indoor and outdoor environments because only these pollutants had legal reference limit values recommended by Indoor Air Quality Control in Public-use Facilities, etc. Act enacted by the Ministry of Environment (MoE), Republic of Korea. The rest of the 37 types of VOCs were excluded from this study because no legal limit value was established for their presence in newly-built residential buildings. Five concentrations (5, 10, 20, 50, and 100 ng/L) of VOCs in a standard solution were estimated using the calibration curve method. Collected samples were quantitatively analyzed by GC/MS (GC-2010, Shimadzu, Japan) connected with a thermal desorber (STD1000, DANI, Italy) using EPA method 8260B. Heated gases were carried to thermally desorb targeted VOCs samples through column VB-1ms (60m × 25mm × 1.0μm) with helium (He) as an inert gas at a flow rate of 100 ml/min for 15 min at 300 °C. Using the external standard method, a toluene calibration curve was applied to quantify TVOC concentrations. Calibration curves of VOCs showed good linearity with high correlation coefficients (r^2^>0.99) (data not shown). Each substance of VOC was also quantified using response factor (RF) values on a working standard (WS) and total ion chromatogram (ITC) for peak areas of VOCs. Then 2,4-DNPH derivatives reacted with HCHO were fixed on solid phase extraction (SPE) vacuum manifolds (VISIPREP, Supelco, USA) and extracted using HPLC-grade 5ml acetonitrile (J. T. Baker, USA) at a flow rate of 5ml/min. Extracted HCHO samples were stored in 50ml brown vials sealed by Teflon tape and place in a refrigerator below 4°C. Extracted 2,4-DNPH derivatives were quantitatively analyzed by high performance liquid chromatography (HPLC, LC-10Avp, Shimadzu, Japan) equipped with an ultraviolet-visible spectroscopy (UV-VIS) detector at a maximum wavelength of 360nm using EPA method 8315A. To ensure the reliability of measured values, we determined a breakthrough capacity of Tenax-TA and 2,4-Dinitrophenylhydrazine (DNPH) cartridges, laboratory and field blank, recovery of instrument reproducibility and thermal desorption, and method detection limits (MDL). The MDL values of target chemicals for GC/MS and HPLC analysis methods were determined by performing at least seven repetitive analyses, and the standard deviation of measured concentrations of each substance was then used to calculate MDL values. The MDL values of each pollutant were shown in Table 1.

**Table 1:** Summary statistics of indoor and outdoor VOCs and HCHO levels (μg/m^3^).

Pollutant	Indoor		Outdoor		Indoor/Outdoor	Limit value^2^	*p-value*^3^
	N	GM ± GSD^1^	N	GM ± GSD			
TVOC	634	230.7 ± 1.7	161	137.8 ± 1.9	1.67	400	0.002
Benzene (MDL : 0.86)	590	3.6 ± 2.4	150	3.0 ± 2.3	1.20	30	0.767
Toluene (MDL : 1.92)	634	22.8 ± 2.1	159	16.5 ± 2.6	1.38	1,000	0.107
Ethylbenzene (MDL : 1.27)	626	4.3 ± 1.9	149	3.2 ± 1.9	1.34	360	0.259
m.p-Xylene (MDL : 1.78)	624	7.2 ± 1.9	149	5.2 ± 1.9	1.38	700	0.512
Styrene (MDL : 0.88)	566	3.7 ± 1.8	116	2.9 ± 1.9	1.28	300	0.482
o-Xylene (MDL : 0.93)	557	2.8 ± 1.8	107	2.2 ± 1.7	1.27	700	0.620
HCHO (MDL : 1.75)	698	20.1 ± 1.6	195	8.1 ± 1.9	2.48	210	0.044

^1^ Geometric mean and geometric standard deviation

^2^ Limit values were recommended under Indoor Air Quality Control In Public Use Facilities, etc. Act (enacted by Ministry of Environment, 2016)

^3^ Statistical analysis by *Student t-test*

### Statistical analysis

In order to compare concentration levels of collected samples for VOCs and HCHO within ECCs and between indoor and outdoor environments, we performed statistical analyses. In addition, SPSS software version 19.0 (IBM, Armonk, New York, USA) was used to determine environmental factors affecting VOC and HCHO levels. All VOC and HCHO samples were log-transformed, since data were right-skewed. The Anderson-Darling normality test was then performed to determine if the dataset of air sampling was log-normally distributed by drawing lognormal probability plots. Student’s t-test was performed to compare geometric means of VOCs and HCHO levels between indoor and outdoor environments at a significance level of 0.05. Using Tukey’s post-hoc test, one-way analysis of variance (ANOVA) was carried out to determine if there was any significant difference between geometric means of VOCs and HCHO levels by environmental factor, including location of center, type of paint used, wooden furniture used, and construction year. To identify environmental factors with significant effect on the increase of VOCs and HCHO levels at ECCs at the same significance level (*p*<0.05), multiple regression analysis was also performed.

## Results

Geographical and environmental characteristics of the 30 ECCs are summarized in Fig 1 and Table 2. Twenty of the 30 ECCs were located in rural areas of Gyeonggi province. They were mostly medium-sized facilities (30 to 317 occupants) with low levels of nearby traffic. The rest of ECCs were large facilities (318 to 600 occupants) with high traffic in urban area (i.e. Seoul metropolitan city) of Korea.

**Table 2:** The environmental characteristics of 30 ECCs selected in this study.

No	Location	Geographic coordinate	Const-ruction year	Area (m^2^)	Occu-pancy (n)	Traffic	Construction material	Interior material	
								Floor	Wall
G-1	Rural	37.489905, 126.703644	2005	1,818	58	High	Cement	Wood	Paint
G-2	Rural	37.626860, 126.704603	2002	1,662	70	Low	Cement	Floor paper^1^	Paint
G-3	Rural	37.700937, 126.832478	2005	1,388	50	Low	Cement	Floor paper	Wallpaper^1^
G-4	Rural	37.899013, 127.199350	2003	2,200	85	High	Cement	Floor paper	Paint
G-5	Rural	37.988970, 127.439735	2000	2,094	100	Low	Cement	Floor paper	Wallpaper
G-6	Rural	37.830420, 126.806392	2001	3,615	58	Low	Cement	Floor paper	Wallpaper
G-7	Rural	38.127958, 127.404380	1988	1,535	52	High	Wood	Floor paper	Wallpaper
G-8	Rural	37.930414, 126.855498	1998	3,170	65	Low	Cement	Floor paper	Paint
G-9	Rural	37.533163, 127.467984	1993	4,706	317	Low	Cement	Floor paper	Wallpaper
G-10	Rural	37.375191, 127.044155	1992	2,111	50	High	Wood	Floor paper	Wallpaper
G-11	Rural	37.392618, 126.854725	2005	2,189	36	Low	Cement	Floor paper	Paint
G-12	Rural	37.389716, 126.994908	2004	3,460	100	Low	Cement	Floor paper	Paint
G-13	Rural	37.501636, 127.147956	1986	2,846	50	Low	Wood	Floor paper	Paint
G-14	Rural	37.443791, 127.139265	2002	2,269	68	Low	Cement	Floor paper	Paint
G-15	Rural	37.396148, 127.297378	1995	5,866	85	Low	Cement	Floor paper	Wallpaper
G-16	Rural	37.292211, 127.145927	2004	4,762	111	Low	Cement	Floor paper	Paint
G-17	Rural	37.302299, 127.162854	1989	1,862	50	Low	Wood	Floor paper	Paint
G-18	Rural	37.225407, 127.196260	2000	757	30	Low	Cement	Floor paper	Paint
G-19	Rural	37.222256, 127.498400	2004	1,630	54	Low	Cement	Floor paper	Paint
G-20	Rural	37.222137, 127.498282	1991	1,962	48	Low	Wood	Floor paper	Paint
S-1	Urban	37.600650, 127.041565	1996	1,096	73	High	Cement	Floor paper	Wallpaper
S-2	Urban	37.553841, 127.025357	2005	10,401	150	High	Cement	Floor paper	Wallpaper
S-3	Urban	37.583168, 126.986726	2000	10,362	170	High	Cement	Wood	Paint
S-4	Urban	37.648810, 127.073555	2006	5,513	165	High	Cement	Floor paper	Paint
S-5	Urban	37.523455, 127.048676	1978	2,434	99	High	Wood	Floor paper	Wallpaper
S-6	Urban	37.527970, 127.126228	2004	3,080	80	High	Cement	Floor paper	Wallpaper
S-7	Urban	37.559204, 127.083755	2003	3,431	600	High	Cement	Floor paper	Paint
S-8	Urban	37.662552, 127.039363	1995	6,695	305	High	Cement	Floor paper	Paint
S-9	Urban	37.680530, 127.056771	1983	1,168	103	Low	Wood	Wood	Paint
S-10	Urban	37.639263, 126.937978	2005	3,496	100	Low	Cement	Floor paper	Paint

^1^ It is one of the traditional Korean interior material (Hanji) which is used to cover floors and walls of the room.

Table 1 contains the results of summary statistics for indoor and outdoor VOCs and HCHO levels. Geometric means (GMs) and geometric standard deviations (GSDs) of TVOC showed significant differences between indoor (230.7±1.7 μg/m^3^) and outdoor (137.8±1.9 μg/m^3^) levels (*p*<0.05), with an I/O ratio of 1.67. Among VOCs, toluene showed the highest levels (22.8±2.1 μg/m^3^ for indoor and 16.5±2.6 μg/m^3^ for outdoor environment). However, there was no statistically significant difference between indoor and outdoor levels of toluene. The rest of VOCs, including benzene, ethylbenzene, styrene, and o,m,p-xylene, showed no difference between indoor and outdoor levels, although their I/O ratios were higher than 1 (ranging from 1.20 to 1.38). The GM of indoor HCHO level (20.1±1.6 μg/m^3^) was significantly higher than that of the outdoor level (8.1±1.9 μg/m^3^), with an average I/O ratio of 2.48 (*p*<0.05). There was also a significant difference in indoor HCHO levels among three rooms of ECCs, and GMs and GSDs were 20.9±1.6 μg/m^3^ for the bedroom, 19.3±1.7 μg/m^3^ for the living room, and 18.2±1.5 μg/m^3^ for the dining room, respectively (*p*<0.05).

According to Table 3, all indoor VOCs levels were significantly higher than outdoor levels in rural area, regardless of traffic (low or high) (*p*<0.01). Likewise, most indoor levels of TVOC, styrene, o-xylene were significantly higher than outdoor levels in urban areas with high traffic (*p*<0.01). In areas of low traffic, most indoor VOCs (except benzene) levels were higher than outdoor levels in urban areas, but the difference was not statistically significant. However, all indoor HCHO levels were significantly higher than outdoor levels in both rural and urban areas, regardless of traffic. Table 4 shows that most indoor VOCs and HCHO levels were significantly higher than outdoor levels (*p*<0.01) by season, in particular, and in the summer (July), in conditions of high temperature and relative humidity, the indoor and outdoor levels of air pollutants were higher than in other seasons.

**Table 3:** The mean levels (GM±GSD) of indoor and outdoor VOCs and HCHO by location and traffic (μg/m^3^).

Pollutant	Rural area										Urban city									
	Traffic (low)					Traffic (high)					Traffic (low)					Traffic (high)				
	N	Outdoor (GM±GSD) ^1^	N	Indoor (GM±GSD) ^1^	*p*-value^2^	N	Outdoor (GM±GSD) ^1^	N	Indoor (GM±GSD) ^1^	*p*-value^2^	N	Outdoor (GM±GSD) ^1^	N	Indoor (GM±GSD) ^1^	*p*-value^2^	N	Outdoor (GM±GSD) ^1^	N	Indoor (GM±GSD) ^1^	*p*-value^2^
TVOC	79	139.4±1.9	307	228.9±1.6	<0.01	25	116.1±1.8	112	235.5±1.6	<0.01	8	181.2±1.6	24	229.9±1.3	0.20	49	132.8±1.9	191	230.9±1.9	<0.01
Benzene	80	0.8±28.1	283	3.1±2,5	<0.01	25	1.5±19.7	102	4.4±1.9	0.04	8	5.6±1.6	23	5.3±1.5	0.78	49	2.6±5.4	182	4.0±2.4	0.08
Toluene	80	11.4±11.7	307	23.1±2,2	<0.01	25	15.5±2.2	112	23.8±2.2	0.02	8	22.7±2.0	24	24.1±1.4	0.82	49	16.2±2.2	191	21.6±2.0	0.02
Ethylbenzene	80	1.2±19.5	306	4.0±1.8	<0.01	25	1.2±17.5	111	4.1±1.8	0.03	8	4.6±1.8	24	5.2±1.4	0.60	49	1.5±20.0	185	5.0±2.0	0.01
m.p-Xylene	80	1.4±31.9	304	6.4±1.7	<0.01	25	1.9±20.3	111	7.4±2.0	0.02	8	7.9±1.7	24	8.3±1.5	0.75	49	3.6±10.2	185	8.6±2.1	0.01
Styrene	80	0.3±72.9	290	3.8±1.9	<0.01	25	0.1±128.7	98	3.2±1.7	<0.01	8	0.3±142.2	20	4.0±1.6	0.18	49	0.1±153.2	158	3.8±1.9	<0.01
o-Xylene	80	0.1±112.8	270	2.4±1.6	<0.01	25	0.0±158.8	96	2.9±1.8	<0.01	8	2.5±1.6	24	2.8±1.4	0.39	49	0.2±110.9	167	3.6±1.9	<0.01
HCHO	91	7.1±2.0	338	18.3±1.6	<0.01	32	8.1±1.8	120	20.7±1.4	<0.01	8	9.8±1.3	24	20.2±1.3	<0.01	64	9.7±1.7	215	23.1±1.8	<0.01

^1^ Geometric mean and geometric standard deviation

^2^ Statistical analysis by *Student t-test*

**Table 4:** The indoor and outdoor VOCs and HCHO levels and environmental factors by season (μg/m^3^).

Season		TVOC		Benzene		Toluene		Ethylbenzene		m.p-Xylene		Styrene		o-Xylene		HCHO		Temp (°C)	Relative humidity (%)
		N	GM ± GSD	N	GM ± GSD	N	GM ± GSD	N	GM ± GSD	N	GM ± GSD	N	GM ± GSD	N	GM ± GSD	N	GM ± GSD		
January (winter)	Indoor	164	230.9 ± 2.1	165	4.1 ± 0.4	161	26.3 ± 1.3	163	4.5 ± 0.5	161	7.6 ± 0.7	165	2.9 ± 0.3	161	2.4 ± 0.3	178	20.0 ± 1.0	23.7	65.4
	Outdoor	49	127.2 ± 1.9	50	2.9 ± 1.7	50	18.5 ± 1.2	50	2.4 ± 0.2	50	4.1 ± 0.4	50	1.6 ± 0.2	50	1.1 ± 0.1	52	7.0 ± 0.6	1.1	63.1
	*p*-value		<0.01		<0.01		<0.01		<0.01		<0.01		<0.01		<0.01		<0.01	-	-
April (spring)	Indoor	158	238.4 ± 2.0	158	2.9 ± 0.4	157	26.3 ± 1.2	158	4.9 ± 0.4	158	9.1 ± 0.6	158	4.0 ± 0.3	158	3.0 ± 0.2	172	19.0 ± 0.9	24.2	66.8
	Outdoor	43	166.0 ± 2.0	43	2.9 ± 1.8	43	18.1 ± 1.1	43	3.9 ± 0.4	43	6.8 ± 0.7	43	3.5 ± 0.4	43	1.7 ± 0.2	51	8.3 ± 0.7	8.2	73.2
	*p*-value		<0.01		0.89		<0.01		< 0.01		<0.01		0.02		<0.01		<0.01	-	-
July (summer)	Indoor	161	311.1 ± 2.2	165	5.9 ± 0.5	160	25.5 ± 1.3	162	4.3 ± 0.4	163	7.2 ± 0.7	157	5.4 ± 0.5	163	2.6 ± 0.2	166	26.5 ± 1.1	26.3	74.6
	Outdoor	21	222.8 ± 2.1	24	5.2 ± 2.0	23	25.2 ± 1.4	24	3.6 ± 0.3	23	6.3 ± 0.6	22	4.5 ± 0.3	24	2.2 ± 0.1	37	13.8 ± 0.8	23.4	82.1
	*p*-value		<0.01		0.03		0.33		0.02		<0.01		<0.01		<0.01		<0.01	-	-
October (autumn)	Indoor	144	244.5 ± 1.9	143	4.4 ± 0.4	143	29.9 ± 1.3	138	5.8 ± 0.6	142	9.2 ± 0.8	142	2.3 ± 0.2	141	2.9 ± 0.4	169	22.5 ± 1.0	24.7	69.4
	Outdoor	45	145.9 ± 1.8	45	4.2 ± 1.8	42	25.5 ± 1.3	42	3.6 ± 0.5	43	5.2 ± 0.7	43	1.4 ± 0.1	43	1.5 ± 0.2	54	9.8 ± 0.8	18.6	72.5
	*p*-value		<0.01		0.26		<0.01		<0.01		<0.01		<0.01		<0.01		<0.01	-	-

Indoor levels of some VOCs (benzene, toluene, ethylbenzene, o,m,p-xylene) and HCHO significantly differed by environmental factor such as the use of carpet on floors, paint on the walls, and wooden furniture (mostly bed frames) at ECCs (*p*<0.05) (Table 5). Indoor levels of some VOCs (ethylbenzene, styrene, o-xylene, and TVOC) and HCHO collected at ECCs constructed before the year 2000 were significantly lower than those at ECCs constructed after 2000 (*p*<0.05). Indoor levels of benzene, toluene, and m,p-xylene at ECCs constructed after the year 2000 were not significantly different from those at ECCs constructed before 2000 (Table 6).

**Table 5:** Comparison of indoor VOCs and HCHO levels by environmental factor (use of carpet, paint, and wooden furniture) (μg/m^3^).

Environmental factor		TVOC		Benzene		Toluene		Ethylbenzene		m.p-Xylene		Styrene		o-Xylene		HCHO	
		N	GM ± GSD	N	GM ± GSD	N	GM ± GSD	N	GM ± GSD	N	GM ± GSD	N	GM ± GSD	N	GM ± GSD	N	GM ± GSD
Carpet	No use	613	228.6 ± 1.7	570	3.7 ± 2.4	227	22.4 ± 2.1	223	4.3 ± 1.8	222	7.1 ± 1.9	201	3.7 ± 1.9	196	2.7 ± 1.7	242	19.9 ± 1.6
	Use	21	300.6 ± 1.8	20	3.4 ± 2.3	306	37.6 ± 2.3	303	6.2 ± 2.0	302	12.6 ± 2.5	273	3.1 ± 1.4	270	5.2 ± 1.9	342	28.8 ± 1.9
	*p*-value		0.01		0.47		<0.01		0.01		<0.01		0.21		<0.01		<0.01
Paint	No use	173	211.4 ± 1.7	20	3.5 ± 2.4	184	18.9 ± 1.9	183	3.6 ± 1.8	183	6.0 ± 2.9	161	3.2 ± 1.5	155	2.5 ± 1.7	190	18.2 ± 1.6
	Use	404	238.0 ± 1.6	372	3.6 ± 2.5	404	23.0 ± 2.0	397	4.4 ± 1.7	395	7.0 ± 2.2	361	3.6 ± 1.9	367	2.7 ± 1.6	460	20.0 ± 1.6
	*p*-value		<0.01		0.16		0.03		<0.01		<0.01		0.41		0.25		<0.01
Wooden Furniture	No use	481	229.0 ± 1.7	448	3.8 ± 2.3	481	21.2 ± 2.1	476	4.3 ± 1.9	475	7.2 ± 1.9	428	3.5 ± 1.8	424	2.8 ± 1.8	528	18.1 ± 1.6
	Use	149	221.5 ± 2.0	138	3.0 ± 2.7	149	23.9 ± 2.2	146	4.4 ± 1.8	145	7.3 ± 1.9	134	4.0 ± 2.0	129	2.8 ± 1.7	162	20.4 ± 1.7
	*p*-value		0.72		0.05		0.04		0.95		0.92		<0.01		0.65		<0.01

**Table 6:** Comparison of indoor VOCs and HCHO levels by construction year.

Name of pollutant	Construction year	N	GM^1^	AM ± SD^2^	*p-value*
TVOC	Before 2000	251	219.37	248.54±129.18	0.03
	After 2000	383	238.42	274.33±154.64	
Benzene	Before 2000	237	3.55	4.59±2.54	0.36
	After 2000	353	3.71	4.81±2.91	
Toluene	Before 2000	251	21.03	27.99±25.48	0.11
	After 2000	383	24.00	31.40±26.30	
Ethylbenzene	Before 2000	249	3.94	4.88±4.26	0.03
	After 2000	377	4.61	5.58±3.90	
m.p-Xylene	Before 2000	249	6.90	8.92±9.40	0.80
	After 2000	375	7.48	9.08±6.10	
Styrene	Before 2000	216	3.20	3.85±2.99	0.01
	After 2000	350	3.98	5.07±5.28	
o-Xylene	Before 2000	227	2.52	3.07±2.54	0.04
	After 2000	330	3.02	3.48±2.10	
HCHO	Before 2000	267	18.33	20.49±10.20	<0.01
	After 2000	431	21.34	24.17±12.67	

^1^GM : Geometric mean;

^2^Arithmetic mean and Standard deviation

In multiple regression analysis, indoor VOCs levels were significantly associated with the use of carpet and paint at ECCs (r^2^ = 0.21) after adjusting for temperature, relative humidity, and ventilation rates (*p*<0.01). Likewise, indoor HCHO levels were significantly associated with the same micro-environmental factors, such as the use of carpets and paints at the ECCs (r^2^ = 0.35) (*p*<0.05) (Table 7).

**Table 7:** Multiple regression analysis for dependent (VOCs and HCHO) and independent variables (use of carpet, paint and wooden furniture).

Dependent	Independent	β	SE	*p-value*
VOCs (N = 323) (r^2^ = 0.21)	Use of carpet	0.12	28.66	<0.01
	Use of paint	-0.11	11.81	<0.01
HCHO (N = 425) (r^2^ = 0.35)	Use of carpet	0.17	2.27	<0.01
	Use of paint	-0.10	0.97	0.02
	Use of wooden furniture	-0.03	0.02	0.07

## Discussion

Results of this study revealed that indoor levels of VOCs and HCHO at 30 ECCs located in Seoul metropolitan city and Gyeonggi province of Korea were significantly higher than those of their outdoor levels. For the elderly population who spent a great deal more time in these facilities than in outdoor environments, exposure to these air pollutants could cause adverse health effects on their pulmonary and respiratory systems. However, geometric means of VOCs and HCHO were significantly lower than legal limit values (400 μg/m^3^ for TVOC, 30 μg/m^3^ for benzene, 1,000 μg/m^3^ for toluene, 360 μg/m^3^ for ethylbenzene, 700 μg/m^3^ for o,m,p-xylene, 300 μg/m^3^ for styrene, and 210 μg/m^3^ for HCHO) suggested by the Indoor Air Quality Act. Act enacted by the MoE in 2006. Regardless of traffic, most indoor VOCs and HCHO levels were significantly higher than outdoor levels in both rural and urban areas. Furthermore, I/O ratios were 1.67 for TVOC and 2.48 for HCHO, indicating that these pollutants were mainly emitted from indoor sources, such as carpets, paints, and new wooden furniture at ECCs [22].

There have been several studies that have examined environmental exposures and evaluated indoor levels of personal exposure to VOCs and HCHO at many residential buildings, elderly care centers, and convalescent hospitals in several countries. The US EPA has reported that indoor levels are approximately 2.5 times higher than outdoor VOCs levels. Clarisse et al. have also reported that indoor HCHO levels are significantly increased by environmental factors, including the introduction of new furniture, the installation of new flooring materials, and remodeling of residential buildings [14, 23, 24]. Katsoyiannis et al. have also conducted an experimental study to assess indoor VOCs and HCHO levels in chambers with or without carpets and found that various types of carpets could increase indoor pollution levels, ranging from 10 to 1,000 μg/m^3^ [25]. Sim et al. have reported that the elderly who live in newly-constructed residential buildings in Korea could be exposed to high concentrations of VOCs and HCHO emitted from construction materials in indoor environments due to insufficient ventilation rates on low or middle floors of such buildings [26]. Results from past studies have revealed that exposure to indoor VOCs and HCHO from various indoor emission sources, such as wooden furniture, carpets, and wall materials, could be higher than those in outdoor environments due to insufficient ventilation rates. This could adversely affect health conditions.

Bentayeb et al. showed that chronic exposure to low-level indoor air pollutants, including CO, NO_2_, PM_0.1_, PM_10_ and HCHO, affected the increasing incidence of respiratory and cardiovascular diseases in the elderly population permanently living in nursing homes in seven European countries [27]. Tunsaringkarn et al. also found that among the elderly in Bangkok, Thailand, indoor levels of HCHO and VOCs were significantly associated with the increase of cancer risks and respiratory symptoms [28]. Mendes et al. also reported that with regard to such air pollutants as CO, NO_2_, PM_2.5_, PM_10_, TVOC, and HCHO indoor levels were significantly higher than outdoor levels. The authors identified that those air pollutants were emitted from indoor sources in their building materials. They also suggested that it is necessary to install adequate control measures (local exhaust ventilation) to reduce long-term exposure to indoor pollutants so that adverse effects among the elderly in Portugal can be prevented [29]. In Korea, Kim et al. reviewed the effects of environmental exposure to indoor air pollutants (NO_2_, SO_2_, O_3_, PM_10_ and VOCs) on the respiratory health of susceptible populations [18]. Findings by studies in various countries suggest that the risk of respiratory and cardiovascular diseases among susceptible populations, including the elderly, were significantly associated with exposure to low-levels of air pollution, primarily emitted from sources in their indoor residences, not outdoor environments.

Like results from previous studies, this study also found that indoor VOCs and HCHO levels differed by construction year, with a cut-off year of 2000, a median of the 1978–2006 period [30–32]. Much higher levels of indoor air pollutants were found in newly-constructed buildings than older buildings. This might be due to the existence of new wooden furniture purchased or new construction materials used [26, 33]. Lim et al. have reported that old elementary schools had much lower HCHO levels than recently-constructed schools when dividing schools into four groups with different time periods: 1 to 10 years, 11 to 20 years, 21 to 40 years, and over 40 years after completion of building construction. Elementary schools constructed 1 to 10 years ago had the highest HCHO levels (27.83 μg/m^3^). However, indoor HCHO level significantly declined as the time period increased [34]. The present study also showed that the year of construction was significantly associated with the decline of indoor pollution levels at ECCs in Korea.

We observed that most indoor VOCs and HCHO levels at ECCs were higher than outdoor levels, regardless of traffic (high or low) in rural and urban areas. In urban areas with high traffic, indoor VOCs (except benzene) and HCHO levels were significantly higher than outdoor pollution levels. However, in areas of low traffic, indoor VOCs (except benzene) levels were higher than outdoor levels, though the difference was not statistically significant. Furthermore, in areas with low traffic, outdoor VOCs and HCHO levels were higher than in areas with higher traffic, indicating that traffic did not affect outdoor pollution levels. In rural areas with high traffic, most outdoor VOCs and HCHO levels were higher than in areas with low traffic, but all indoor VOCs and HCHO levels were higher than outdoor pollution levels, indicating that VOCs and HCHO must be emitted from the indoor environments of ECCs, regardless of traffic. The evidence implied that principle environmental factors affecting VOCs and HCHO levels must be indoor sources (carpets, paint, and wooden furniture) at the ECCs in Korea.

This study has several strengths. First, this is the first study that characterizes both indoor VOCs and HCHO levels at 30 ECCs located in Seoul metropolitan city and Gyeonggi province in Korea. I/O ratio was also calculated for each pollutant. All I/O ratios ranged from 1.20 to 1.67 for VOCs and 2.48 for HCHO, indicating that these pollutants came from indoor emission sources rather than outdoor environments. Second, our study revealed significant differences in indoor HCHO levels among three rooms of ECCs (bedroom > living room > dining room). Furthermore, indoor pollution levels were compared by environmental factors to identify whether there was any significant difference by factor (e.g., use of carpet, paint, wooden furniture, and construction year). We found that indoor VOCs and HCHO levels were significantly different by those environmental factors.

Despite its several strengths, this study has limitations. First, we could not collect a large enough number of air monitoring samples to represent all ECCs in the urban and rural areas of Korea. In addition, we did not extend our study to other ECCs in other regions of Korea. Furthermore, due to the limited number of field technicians and time, sampling devices, and analytical tools in our laboratory, we did not fully analyze daily or monthly variations in VOCs or HCHO levels in indoor and outdoor environments. Most importantly, we could not quantitatively estimate statistical values on health outcomes or risks (e.g. Odds ratio, etc.) among the elderly population, though the significant increase in indoor levels of VOCs and HCHO exposures were observed at ECCs. Therefore, we could not investigate the association between indoor exposure to air pollutants and adverse health effects. However, many peer-reviewed literatures demonstrated that low-level exposures to indoor VOCs and HCHO are significantly associated with the increase of health problems, including asthma and other respiratory and cardiovascular diseases. Further studies are needed to investigate the effects of long-term exposures to low indoor VOCs and HCHO levels on chronic health outcomes among the elderly population in Korea.

## Conclusion

Indoor VOCs and HCHO levels from indoor emission sources (carpet, paint, and wooden furniture) at thirty ECCs located in urban and suburban areas of Korea were significantly higher than those in outdoor environments. Indoor emission sources significantly affected their levels in different rooms of ECCs. However, we did not investigate adverse health effects resulting from chronic exposure to low levels of these air pollutants among the elderly population who spent most of their daily time (>90%) at their ECCs. Therefore, further studies are needed to determine indoor emission sources with the increase in indoor VOCs and HCHO levels and to identify potential health effects on the elderly that might arise due to exposure to increased levels of VOCs and HCHO at different ECCs located in different regions of Korea.

## Supporting information

S1 Table (XLS)
